# Supplementary material for: The Systems Biology Research Tool: evolvable open-source software
Source: BMC Syst Biol. 2008 Jun 29;2:55. doi: 10.1186/1752-0509-2-55 (PMC2446383; doi:10.1186/1752-0509-2-55)
Supplement: Additional file 1 — SBRT Archive. An archive of the current version of the Systems Biology Research Tool. [file 1752-0509-2-55-S1.zip › sbrt-1.4.0/doc/developers_guide/api/sbrt/kernel/math/class-use/ExceededLimitException.html]

Uses of Class sbrt.kernel.math.ExceededLimitException


|  |  |  |
| --- | --- | --- |
| **Overview** \| **Package** \| **Class** \| **Use** \| **Tree** \| **Deprecated** \| **Index** \| **Help** | |  |
| PREV   NEXT | **FRAMES**    **NO FRAMES**     **All Classes** |  |


---


## **Uses of Class sbrt.kernel.math.ExceededLimitException**

| Packages that use ExceededLimitException | |
| --- | --- |
| **sbrt.shell.mng.fba** | Provides classes and interfaces for creating Flux Balance Analysis process managers. |
| **sbrt.shell.mng.geom** | Provides classes and for creating geometry process managers. |

Uses of ExceededLimitException in sbrt.shell.mng.fba

| Methods in sbrt.shell.mng.fba with parameters of type ExceededLimitException | |
| --- | --- |
| `static ApplicationException` | `CdHarFluxVectorManager.interpret(ExceededLimitException e)`             Returns an `ApplicationException` whose message describes the error that ocurred. |

Uses of ExceededLimitException in sbrt.shell.mng.geom

| Methods in sbrt.shell.mng.geom with parameters of type ExceededLimitException | |
| --- | --- |
| `static ApplicationException` | `CdHarManager.interpret(ExceededLimitException e)`             Returns an `ApplicationException` whose message describes the error that ocurred. |

---


|  |  |  |
| --- | --- | --- |
| **Overview** \| **Package** \| **Class** \| **Use** \| **Tree** \| **Deprecated** \| **Index** \| **Help** | |  |
| PREV   NEXT | **FRAMES**    **NO FRAMES**     **All Classes** |  |


---
